# GNAQ mutations drive port wine birthmark-associated Sturge-Weber syndrome: A review of pathobiology, therapies, and current models

**DOI:** 10.3389/fnhum.2022.1006027

**Published:** 2022-11-03

**Authors:** William K. Van Trigt, Kristen M. Kelly, Christopher C. W. Hughes

**Affiliations:** ^1^Department of Molecular Biology and Biochemistry, School of Biological Sciences, University of California, Irvine, Irvine, CA, United States; ^2^Department of Dermatology, School of Medicine, University of California, Irvine, Irvine, CA, United States

**Keywords:** GNAQ, guanine nucleotide binding protein alpha subunit q, Gα_*q*_, port wine birthmark, Sturge-Weber syndrome, brain vascular malformation, capillary malformation

## Abstract

Port-wine birthmarks (PWBs) are caused by somatic, mosaic mutations in the G protein guanine nucleotide binding protein alpha subunit q (GNAQ) and are characterized by the formation of dilated, dysfunctional blood vessels in the dermis, eyes, and/or brain. Cutaneous PWBs can be treated by current dermatologic therapy, like laser intervention, to lighten the lesions and diminish nodules that occur in the lesion. Involvement of the eyes and/or brain can result in serious complications and this variation is termed Sturge-Weber syndrome (SWS). Some of the biggest hurdles preventing development of new therapeutics are unanswered questions regarding disease biology and lack of models for drug screening. In this review, we discuss the current understanding of GNAQ signaling, the standard of care for patients, overlap with other GNAQ-associated or phenotypically similar diseases, as well as deficiencies in current *in vivo* and *in vitro* vascular malformation models.

## Introduction

**FIGURE 1:**
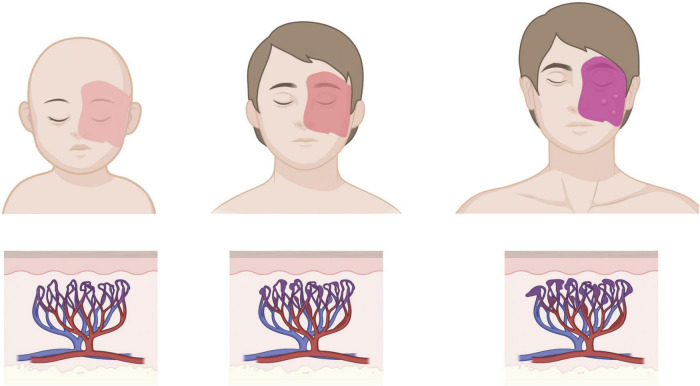
Age progression of cutaneous PWB. Birthmark is usually apparent from birth as a light pink patch that can get darker with age, corresponding to progressive dilation of affected capillaries in the dermis. Patients in adulthood may develop soft tissue hypertrophy or nodularity in the absence of clinical intervention. Images created using BioRender.

Port-wine birthmark (PWB; also known as nevus flammeus and Port Wine Stain) is a congenital, progressive blood vessel disease that manifests as regions of skin that darken and thicken with age (Figure 1; Yin et al., 2017). Approximately one in 350 newborns is born with capillary malformations (CMs) like PWB (Kanada et al., 2012;

Higueros et al., 2017). PWB lesions are usually apparent from birth as a unilateral light pink to red patch typically on the face or neck, although PWB can occur on any area of the body (Martins et al., 2017). CMs that are also found in the eyes and/or the brain are commonly referred to as Sturge-Weber syndrome (SWS). SWS has also been described as an over-arching syndrome with three types – Type I with neurological and skin involvement, with or without glaucoma; Type II with skin, but no neurological involvement, with or without glaucoma; and, Type III neurological only (Sturge Weber Foundation). In this review we will discuss cutaneous CMs as PWB and CMs with neurological involvement as SWS.

**FIGURE 2:**
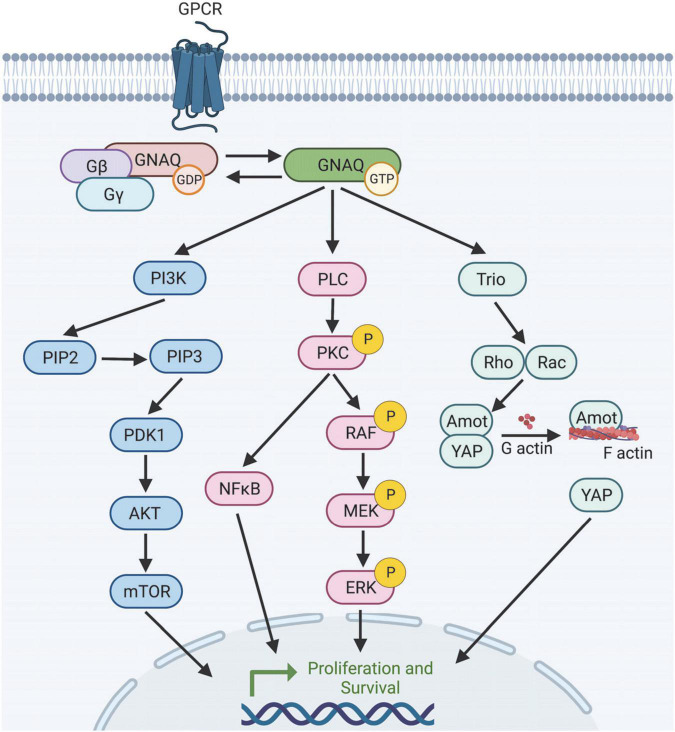
Selection of predicted downstream targets of GNAQ. GNAQ can activate PI3K leading to mTOR activation; stimulate the MAPK pathway, or activate non-canonical Hippo signaling through Rho/Rac. How GNAQ* leads to vessel instability and PWB/SWS disease progression is poorly understood. Image created using BioRender.

The CMs associated with PWB/SWS are caused by a somatic activating mutation in guanine nucleotide binding protein alpha subunit q (GNAQ) (although sometimes found in the paralog GNA11) that results in an arginine to glutamine substitution at the 183 amino acid residue (p.R183Q) (Couto et al., 2016). Disease appears to be manifested by expression of this mutant protein primarily, perhaps exclusively, in endothelial cells (ECs) leading to an increase in proliferation and capillary overgrowth, as summarized in Figure 2. A glutamine to leucine (p.Q209L) GNAQ mutation can also cause CMs and cancer, although this mutation has not yet been reported in PWB patients (Bichsel and Bischoff, 2019; Schneider et al., 2019; Jain et al., 2020). It has been included in this review in discussions of possible overlap with p.R183Q GNAQ constitutive activity. PWB and SWS are typically differentiated from other types of capillary vascular lesions through genetic tests that confirm the presence of mutant GNAQ in these lesions.

Since the mutations are somatically acquired, no difference in disease prevalence is expected or known to occur between sexes or races, although there are discrepancies in diagnosis and treatment.

## Port-wine birthmark

As noted above, PWB lesions can occur anywhere on the body but are particularly prevalent around the head and neck. Without treatment (or with treatment-resistant PWB), cutaneous lesions have the potential to progress with vascular hyperplasia, increasing prevalence of ectatic (dilated) vessels that cause the skin to darken in color (from pink or red to purple), and in some cases nodularity can develop (Yin et al., 2017; Lee et al., 2019). Perivascular cell disorganization is also observed (Couto et al., 2016). Disease progression is slow, however, so patients may not experience serious pathologies until late teens or adulthood.

Nodules are predicted to occur in about 40 percent of untreated PWB, with a mean onset of 22 years, and soft-tissue hypertrophy is seen in about 60% of cutaneous PWB patients (Lee et al., 2015; Higueros et al., 2017). Early laser intervention is thought to significantly delay onset of nodularity, however nodularity and hypertrophy can be difficult to treat with lasers and may require excisional surgical intervention (Tierney and Hanke, 2009). The exact mechanisms leading to hypertrophy and nodularity are not characterized, but Yin et al. (2017) identify upregulation of PP2A, DAG, and activation of PI3K, PKCα, PDPK1, and PLCγ in the patient tissue. This aberrant signaling was mostly detected in the ECs but also had some spillover into surrounding fibroblasts and pericytes proliferating in the stromal tissue, although whether these effects are cell autonomous or due to endothelial-released factors is not known (Yin et al., 2017). However, multilineage detection of mutant GNAQ and aberrant downstream targets in PWB tissue suggest that the somatic mutation may be propagated from a progenitor cell population into several adult cell types, in addition to EC (Couto et al., 2016; Yin et al., 2017). Other researchers have predicted or detected the driving of lesion formation by mutant GNAQ (GNAQ*) through Angiopoietin 2 (ANGPT2), PI3K, and MAPK activation (Shirley et al., 2013; Nakashima et al., 2014; Huang et al., 2017; Cong et al., 2020).

**FIGURE 3:**
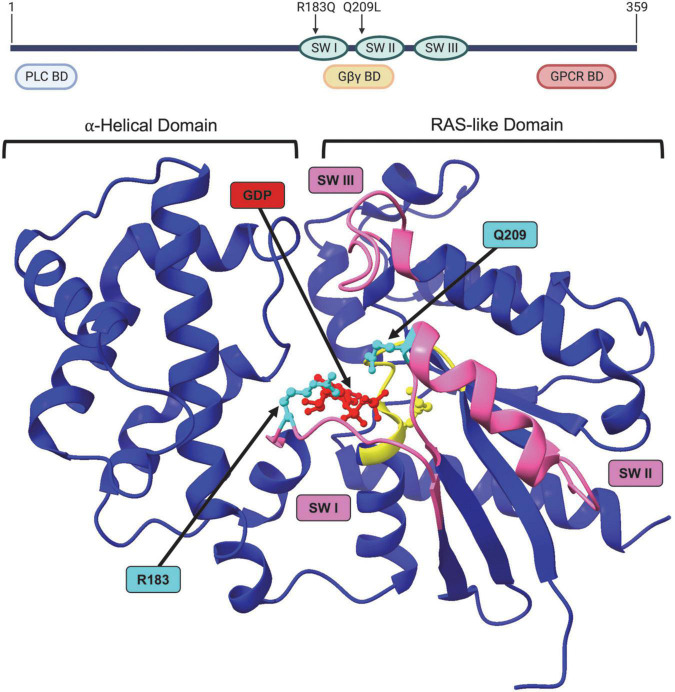
Structure of GNAQ. Protein map of GNAQ. GNAQ has 3 switch regions (SW): switch regions I and II are part of the GTP binding cleft and have a RAS-like domain for GTP hydrolysis; all three switch regions have key sites for extrinsic regulation by G protein signaling modulators. The p.R183Q and p.Q209L activating mutations are present in switch regions I and II, respectively. GNAQ has a binding domain (BD) for PLC effector function, a binding domain that interacts with the Gβγ subunit during the “off” state, and a GPCR binding domain. Image created using BioRender. Crystal structure (PDB ID: 4GNK) with GDP (red) docked in binding cleft and p.R183Q and p.Q209L mutations (cyan). P loop region with Walker A motif shown in yellow. Switch (SW) I, II, and III shown in pink. Supplementary Video available online. Molecular graphics performed with UCSF ChimeraX, developed by the Resource for Biocomputing, Visualization, and Informatics at the University of California, San Francisco, with support from National Institutes of Health R01-GM129325 and the Office of Cyber Infrastructure and Computational Biology, National Institute of Allergy and Infectious Diseases.

Both the p.R183Q and p.Q209L GNAQ mutations are located within the predicted guanine triphosphate (GTP) binding cleft and help stabilize GNAQ affinity for the GTP-bound “on” state (Figure 3). Recent data suggests the p.209 position also plays a role in the switch II domain (discussed later) that helps shield the binding cleft from regulatory proteins (Higueros et al., 2017; Bichsel and Bischoff, 2019). Simultaneous GNAQ and GNA11 mutations are uncommon in patients (Daniels et al., 2012; Schneider et al., 2019). Downstream RAS pathway activation was proposed early on as the causative driver of pathogenesis because it explains how affected cells increase proliferation and inhibit apoptosis (Higueros et al., 2017). GNAQ* cells typically comprise 6–85% of the total ECs in the PWB lesion; the range in heterogeneity correlates with disease severity, with higher ratios of mutant GNAQ cells contributing to increased pathology (Couto et al., 2016; Huang et al., 2017; Martins et al., 2017; Yin et al., 2017; Jordan et al., 2020). Testing for genetic panels usually costs about US $3,000 and are often not covered by insurance (Perelman School of Medicine at the University of Pennsylvania).

## Sturge-Weber syndrome

**FIGURE 4:**
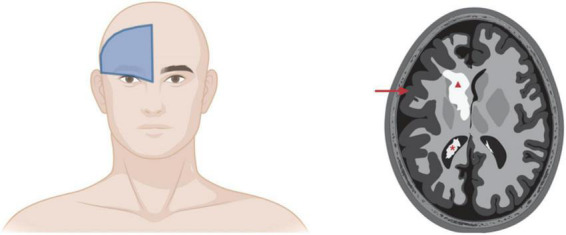
Sturge-Weber syndrome involvement of brain. **Left panel:** Cutaneous PWB in the frontal placode is the best predictor of SWS involvement of eyes and/or brain. **Right panel:** Artistic rendering of SWS complications in brain. Red triangle, cerebral calcification; red arrow, leptomeningeal thickening and cerebral atrophy; red asterisk, enlarged choroid plexus. Images created using BioRender.

Sturge-Weber syndrome was named after William Allen Sturge and Frederick Parkes Weber (Higueros et al., 2017; Martins et al., 2017; Yin et al., 2017). PWB endothelium in what has traditionally been called the ophthalmic V1 trigeminal nerve distribution is associated with SWS, perhaps reflecting the lineage that underwent the somatic mutation in GNAQ during development (Kanada et al., 2012; Higueros et al., 2017). More recent studies have identified PWB cutaneous lesions in the triangular area from the forehead midline, outer edge of the eye, and top of the ear as the best prediction of SWS involvement (Figure 4; Waelchli et al., 2014; Sabeti et al., 2021). This area is called the frontal placode and typically develops its own vasculature derived from the prosencephalon and anterior mesencephalon. SWS affects approximately one in 50,000 individuals (Kanada et al., 2012; Higueros et al., 2017). About 80% of SWS patients have the p.R183Q GNAQ mutation (Shirley et al., 2013; Nakashima et al., 2014; Huang et al., 2017). SWS is characterized by cutaneous PWBs which are usually more extensive than non-syndromic PWB, ophthalmologic impairment, especially glaucoma, and malformed vessels in the thickened leptomeninges (Shirley et al., 2013). These torturous leptomeningeal vessels cause neurological deficits, macrocephaly, seizures, astrocytosis, and cortical atrophy with calcification (Shirley et al., 2013; Huang et al., 2017; Jordan et al., 2020). Dilated deep draining veins can exacerbate SWS pathology. SWS leptomeningeal vessels also have increased fibronectin and VEGF expression and EC proliferation and apoptosis (Higueros et al., 2017). Seizures occur in 75% of patients within the first year of life and 90% of SWS patients will experience seizures before 2 years of age (Higueros et al., 2017). Since the GNAQ mutations in SWS are widely prevalent in the EC compartment, many researchers speculate that the GNAQ*-EC have impaired blood–brain barrier capability and possibly aberrant interactions with surrounding tissue in the leptomeninges and cortex that cause these neurovascular dysfunctions and seizures, possibly due do hypoxia, ischemia, and gliosis (Higueros et al., 2017; Huang et al., 2017).

As noted, there are three different subclasses of SWS that are sometimes referenced in the literature and that vary from each other by the combination of tissues involved. Type I SWS involves cutaneous PWB and brain vascular malformations with or without eye involvement (usually glaucoma). Type I SWS is the most common and usually only affects one side of the brain (Sturge Weber Foundation). Type II involves facial cutaneous PWB and eye involvement but not brain. Type III characterizes SWS with brain vascular malformations without skin PWB or (rarely) eye. Type III can only be diagnosed *via* brain scanning (Sturge Weber Foundation). These categories have not been universally adopted; we and others hypothesize that the type of classification is not useful because treatment decisions are made on an individual level depending on symptoms, patient age, and disease severity (Sabeti et al., 2021).

About half of SWS patients have pathogenesis that affects the eyes (Type I and II) (Bichsel and Bischoff, 2019; Jordan et al., 2020). In this case, a patient may develop choroidal hemangioma, seen as PWB vessel overgrowth in the choroid that leads to thickening of the choroid and increased intraocular and intravenous pressures, eventually causing glaucoma (Bichsel et al., 2019; Jordan et al., 2020). Choroidal hemangioma, unlike infantile hemangioma is not responsive to propranolol treatment. Common treatment includes therapeutics to lower intraocular pressure or enucleation surgery. Only the p.R183Q GNAQ mutation has been detected in choroidal hemangioma patients, although this is probably sampling error from small datasets.

The remainder of this review will focus on GNAQ and its role in driving PWB-associated SWS. We do not intend to be exhaustive but will concentrate on the current literature regarding GNAQ signaling, the role of GNAQ in driving brain CMs, and on illuminating current exciting research on molecular mechanisms and models of disease pathology.

### Diagnosis of Sturge-Weber syndrome

Extracutaneous involvement is suspected with SWS whenever patients have PWB on their forehead, even without the presentation of neurological symptoms. SWS involvement can be evaluated through a variety of imaging techniques that play a crucial role in detection, diagnosis, and follow-up of this disease.

#### Computed tomography

Computed tomography (CT) with or without contrast enhancement is frequently used due to its ability to detect reduced parenchymal brain volume, enlarged ventricles, or enlarged choroid plexus. CT scans can also detect calcification better than X-ray or magnetic resonance imaging (MRI) (Higueros et al., 2017).

#### Magnetic resonance imaging

Gadolinium enhanced MRI is the principle imaging technique used for SWS diagnosis (Higueros et al., 2017). It can effectively identify calcified areas as well as structural and functional anomalies in the leptomeninges, abnormal venous drainage, reduced and/or enlarged brain structures, and hypermyelination underneath the leptomeningeal lesion(s) (Higueros et al., 2017).

#### Perfusion imaging

Perfusion imaging can play a crucial role in identifying stage of disease. Perfusion imaging may be performed in several ways: in conjunction with CT or MRI; or, by using radiotracers and either single photon emission computed tomography (SPECT) or positron emission (PET) techniques. Most SWS lesions tend to be hyperperfused with blood early in childhood, but this tends to change to hypoperfusion later—leading to progressive ischemia, hypoxia, and nutrient starvation of the brain around the vascular malformation (Higueros et al., 2017). It is predicted that this switch to progressive hypoperfusion in SWS is responsible for the neurological degradation characteristic of this disease.

#### Electroencephalogram

Like perfusion imaging, electroencephalogram (EEG) frequently can identify progression to an increasingly abnormal SWS signature correlated with patient age. EEG readings of advanced SWS brain tissue detect epileptiform characteristics with decreased brain voltage and focal discharge in the hemisphere affected by SWS (Higueros et al., 2017).

#### Angiography

Angiographical imaging is not usually performed for patients with SWS unless clinically indicated. Unlike other vascular malformation diseases, SWS lesions are low-flow malformations that are unlikely to undergo thrombotic events. Angiography may be used prior to craniotomy procedures to detect bleeding risk (Higueros et al., 2017).

### Sturge-Weber syndrome treatment

A recent consensus statement prepared by 12 top US expert clinicians in dermatology and SWS identified several key guidelines for the management of PWB associated with SWS (Sabeti et al., 2021).

Early diagnosis of PWB/SWS (as close to birth as possible) and intervention maximizes the success of treatment(s) (Waelchli et al., 2014; Sabeti et al., 2021). Several factors must be weighed when designing a treatment plan, including but not limited to reducing birthmark appearance, diminishing or preventing nodularity and/or soft tissue hypertrophy, minimizing the impact to patient quality of life and self-esteem, and lastly, financial considerations.

#### Cutaneous port-wine birthmarks treatments

Light-based therapy, especially with pulsed-dye laser (PDL), is the standard of care for PWB in the United States (Higueros et al., 2017; Sabeti et al., 2021). Experienced clinicians can safely and effectively use PDL on patients of all ages, including infants. Other wavelengths (532, 755, and 1,064 nm) have been used and are especially useful for treating PDL-resistant PWB lesions. The longer wavelengths are also better for penetrating larger or deeper vessels like those that occur in nodular or hypertrophic lesions, but these laser devices also pose an increased risk of damage to non-target adjacent tissue (Izikson et al., 2009; Sabeti et al., 2021). Laser therapies target heat absorption of hemoglobin and cause photocoagulation, with the goal to remove aberrant vasculature by selective damage and photothermolysis (Bernstein, 2009; Sabeti et al., 2021). PDL therapy has also been combined with other techniques like infrared laser pulses or bipolar radiofrequency to improve efficacy in treatment-resistant PWB (Bae et al., 2017; Sabeti et al., 2021). Pain management is an important concern with laser therapy. Topical or injected local anesthetics, skin cooling, nerve blocks, and/or general anesthesia can be used during treatment (Sabeti et al., 2021).

Pulsed-dye laser treatment response is difficult to predict, but can lighten PWB skin color by 50–70% through at least eight to ten PDL sessions, although complete clearance is rarely achieved and touch-up follow-up treatment is frequently used to maintain level of clearance (Sabeti et al., 2021). There are no consistent guidelines for intervals between treatment sessions. Patients with lighter skin tones tend to have a better PDL treatment response than those with darker skin (Higueros et al., 2017; Sabeti et al., 2021). Patients with darker skin require more skin cooling or adjustment of laser parameters to prevent scarring or blistering. PWB on the face and neck, especially the lateral sides of the face, are easier to treat than PWB on the lower body extremities (Yu et al., 2016; Sabeti et al., 2021). It is possible that patients with SWS have PWB lesions that are more likely to be treatment-resistant, although this has not been clearly established and the mechanism is unknown (Sabeti et al., 2021). As mentioned earlier, PWBs are more effectively treated in younger than older patients, especially if the affected area is still flat without signs of nodularity or hypertrophy.

There are some adjuvant treatments which have been tried with PDL, although none have achieved marked improvement over laser therapy alone and are no longer used frequently in clinical treatments (Wang et al., 2022). Combination of PDL with topical imiquimod demonstrated slightly improved effect in treating PWB over PDL alone in small scale studies through immunomodulation activity that reduces tumor necrosis factor (TNF), interferon-γ (IFN-γ), as well as decreasing pro-angiogenic production of matrix metalloproteinase 9 (MMP9) and increasing apoptosis (Sidbury et al., 2003; Lipner, 2018; Wang et al., 2022).

Rapamycin, an mTOR inhibitor, has been studied in a variety of diseases for its immunosuppressant and antiproliferative activities. Human clinical studies of topical rapamycin combined with PDL on affected areas of the face and neck showed some efficacy in limiting cutaneous vessel regrowth *via* HIF-1α and VEGF inhibition, but results were disappointing with wider clinical use (Lipner, 2018; Wang et al., 2022). Using animal models, PDL and axitinib, an inhibitor of MEK/ERK, have been shown to prevent vessel regrowth (Gao et al., 2015). Efficacy in patients has not yet been demonstrated. A small scale trial with PDL and bosentan (an endothelin receptor antagonist) showed efficacy in one of four PWB patients treated (Taquin et al., 2016).

As laser therapy works through light absorption of hemoglobin, the perfusion of hemoglobin-laden vesicles during PDL treatment has been tried and was shown to increase efficacy of PDL for dilated and deeper vessels in animal models, improving the safety of PDL by reducing the laser intensity required and thereby reducing off-target tissue effects (Rikihisa et al., 2017, 2018). In another preclinical study, PDL therapy with the α1A- and partial α2A-adrenoreceptor agonist oxymetazoline was used for the treatment of erythematotelangiectatic rosacea and showed some efficacy for PDL combination treatment of PWB in mouse models (Kelly et al., 2020). Both of these strategies warrant further investigation.

Photodynamic therapy, unlike PDL, uses perfusion of a photosensitive dye and an excitatory source to produce reactive oxygen species (ROS) that destroy local tissue. This technique is not widely used in the US, but may be beneficial for resistant PWB, and when treating darker skin types as the melanin content of skin does not reduce photodynamic therapy efficacy (Sabeti et al., 2021).

Lastly, surgical intervention may be employed to selectively remove hypertrophic or nodular tissue.

#### Treatments targeted specifically for Sturge-Weber syndrome

Unfortunately, SWS-specific treatments that target neurodegeneration are not currently available. The cornerstone for treatment is anticonvulsant therapy to limit damage due to seizures. Carbamazepine and oxcarbazepine are frequently prescribed to SWS patients, sometimes even prophylactically before the first incident, to prevent epileptic events (Higueros et al., 2017). Low-dose aspirin may additionally help prevent seizures and ischemia in SWS tissue. Seizures are untreatable in about half of SWS patients; in these cases, early surgical intervention with lesionectomy, corpus callosotomy, and/or hemispherectomy may be considered (Higueros et al., 2017). Patients with these invasive brain interventions as well as patients with cognitive deficits or hyperactivity may need physiotherapy, educational therapy, and behavioral therapy. Some studies have shown oral treatment with rapamycin improves cognitive function and recovery time from stroke-like episodes (Sebold et al., 2021). Larger clinical studies are required.

Treatment for glaucoma aims to prevent degeneration of the optic nerve by decreasing intraocular pressure. Placement of a drainage device is often required and aqueous suppressants or medications to increase outflow are usually effective (Higueros et al., 2017).

Sturge-Weber syndrome patients are prone to thyroid diseases and should be routinely examined for growth hormone deficiencies and hypothyroidism (Miller et al., 2006; Bachur et al., 2015).

## GNAQ and the G protein landscape

Heterotrimeric guanine nucleotide-binding proteins (G proteins) transduce a variety of important autocrine and paracrine signals from numerous receptors, such as those for hormones, neurotransmitters, and chemokines. These effect changes in such diverse cell functions as gene transcription, metabolism, cell motility, embryonic and gonadal development, and learning and memory (Neves et al., 2002). Reflecting this diversity of function, the G protein coupled receptor (GPCR) family has over 800 family members, and almost 30% of drug discovery targets influence GPCR signaling (Urtatiz and Van Raamsdonk, 2016).

The G protein complex is made up of three subunits: α, β, and γ. Upon activation of a GPCR, the Gα subunit exchanges guanine diphosphate (GDP) for GTP, dissociating Gα from the Gβγ heterodimer and allowing Gα and Gβγ to transduce downstream signals. There are currently 18 identified Gα, 5 Gβ, and 12 Gγ subunits (Syrovatkina et al., 2016). With respect to receptor and effector specificity, and sequence and functional similarities, the Gα subunit can be further divided into four families: Gα_*i*_, Gα_*s*_, Gα_12_, and Gα_*q*_ (Simon et al., 1991; Neves et al., 2002). The Gα_*i*_ family is the biggest and most diverse group of G proteins and these are expressed in most cells, although there are a few that are specific to neurons, platelets, and rod and cone cells. The i stands for “inhibition,” as a majority of these downstream responses limit activity of cAMP-dependent protein kinases (Syrovatkina et al., 2016). The Gα_*s*_ family (s for “stimulation”) only has two members; Gα_*s*_ is expressed in most cell types while Gα_*olf*_ is only expressed in olfactory sensory neurons. The Gα_12_ family also has two members that are widely expressed. Lastly, the Gα_*q*_ family has four members: GNAQ and its paralog Gα_11_ are ubiquitously expressed, while Gα_14_ and Gα_15/16_ are expressed in soft organs (kidney, lung, and liver) and hematopoietic cells, respectively.

### Gene

In humans, GNAQ is encoded by the GNAQ gene on chromosome 9 at 9q21.2 (Dong et al., 1995). There is also a pseudogene at location 2q21 as well as the paralog Gα_11_ (GNA11) at location 19p13.31 (Dong et al., 1995). The eight-exon GNAQ mRNA transcript is 6,882 nucleotides long, coding for a 359 amino acid protein (Figure 3; NM_002072.5, P50148.4, CCDS6658.1). Although the GNA11 paralog is also 359 amino acids long, there are areas of dissimilarities in protein sequence. These disparities do not occur in the conserved active site amino acids, however, and the GNAQ p.R183Q or p.Q209L amino acid substitutions (c.548G > A and c.626A > T nucleotide substitutions, respectively) in SWS patients can also occur in GNA11 at the same corresponding amino acid positions in GNA11-driven SWS (Dong et al., 1995; Couto et al., 2016; Higueros et al., 2017). GNA11 mutations will not be covered in this review as they cause the same constitutive Gα constitutive activity and SWS disease pathogenesis, albeit GNA11 mutations are detected at lower patient frequency than GNAQ (Jordan et al., 2020).

### Protein

**FIGURE 5:**
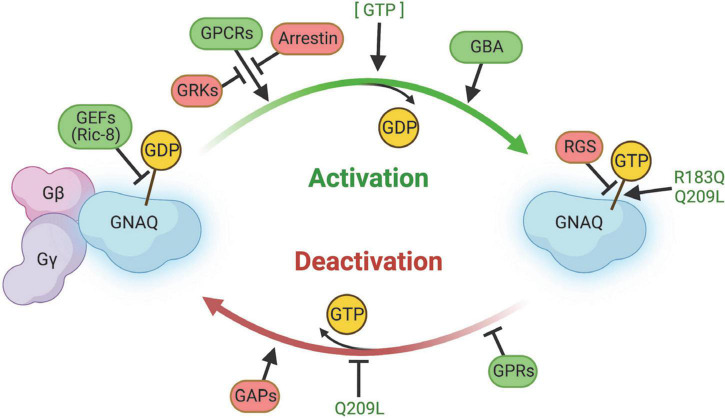
Summary of regulators of GNAQ signaling. GEFs, GPCRs, GBA proteins, and GPRs promote GNAQ-GTP “on” signaling and/or delay GTP hydrolysis. Arrestin, GRKs, RGS, and GAPS slow activation of GNAQ, stimulate GTP hydrolysis, and/or stabilize the GNAQ-Gβγ complex. The p.R183Q and p.Q209L strongly stabilize GNAQ-GTP binding and the p.Q209L mutation impairs intrinsic GNAQ GTPase activity. Image created using BioRender.

G proteins, including GNAQ, function in a GTPase cycle. GPCRs act as a kind of guanine nucleotide exchange factor (GEF) to initiate release of GDP and binding of GTP to GNAQ, causing dissociation of GNAQ from the Gβγ complex (Figure 5; Syrovatkina et al., 2016). GNAQ-GTP is eventually hydrolyzed back to GNAQ-GDP by its intrinsically weak GTPase activity to terminate GNAQ effector function. This process of GTP hydrolysis can be expedited by GTPase activating proteins (GAPs). Re-association of GNAQ with Gβγ terminates the signaling cascade and completes the GTPase cycle.

The amino acid sequence of the GDT/GTP binding cleft is highly conserved (Dong et al., 1995; Syrovatkina et al., 2016). The GNAQ protein has a RAS-like GTPase domain and an α-helical domain, connected by the Linker 1 and Linker 2 domains. The RAS-like GTPase and α-helical domains surround the GTP hydrolysis cleft, thereby protecting the GDP or GTP nucleotide from the surrounding solvent and serving as an inhibitory barrier for regulation by GEFs, GAPs, and the Gβγ complex (Syrovatkina et al., 2016). GDP/GTP binding and hydrolysis take place in the RAS-like domain of GNAQ through a “switch” mechanism. The domain comprises six β-sheets and five α-helices on each side, defining a nucleotide-binding fold, with several key amino acids lining the fold to specify guanine binding (Sprang, 1997; Wolf et al., 1999; Oldham and Hamm, 2006; Syrovatkina et al., 2016). The side loops around the cleft are the switch regions – Switch I and Switch II. The N-terminus of the Gαα-helix and the Switch II region associate with a propellor structure of Gβ in the Gβγ complex (Syrovatkina et al., 2016). Switch I and II together with the P loop region interact with GDP/GTP and the Mg^2+^ coordinating ion. Two of the key sequences in the cleft are the NKKD sequence in Switch II, predicted to form a bifurcated hydrogen bond between aspartate and the guanine base, while the second sequence is a Walker A motif in the P-loop region where the GTGESGKS sequence is associated with binding of the β-phosphate of GDP/GTP (Wolf et al., 1999; Bosch et al., 2012; Syrovatkina et al., 2016). The α-helical domain and switch III regions play a role in G protein regulation (discussed later).

GNAQ has a high affinity for GDP. GEFs (including GPCRs) work to weaken this affinity by causing instability in the protein switch regions. GEFs on their own have a relatively low affinity for GNAQ in either the GDP or GTP bound state. Instead, for the exchange reaction to occur, the GEF must interact with the switch regions in a way that partially displaces the Mg^2+^ ion, which destabilizes bound GDP and causes a push/pull interaction between the switch regions and GDP. The destabilized GDP is released as a stable GEF-GNAQ complex forms, leaving GNAQ available to bind GTP (GTP is usually at higher intracellular concentrations than GDP) (Syrovatkina et al., 2016). More detailed structural and mechanistic information regarding conformational state, the ways in which GEFs initiate interaction with GNAQ, as well as the way GDP is released, still need to be elucidated.

Originally, it was thought that GPCRs diffuse freely within the cell membrane and interact with G proteins though collision, but there are data suggesting un-activated GPCRs in a pre-assembled complex with G proteins: agonist activation of the GPCR then causes a conformational change in the pre-organized complex that facilitates G protein signal transduction (Strange, 2008; Syrovatkina et al., 2016). The different kinds of GPCR and G protein interaction depend on the pair, which may explain the range of GPCR activity in different cell types and tissues. Additionally, GPCR-agonist binding alone is sometimes insufficient to cause G protein activation and may require additional interactions with other proteins. On the other hand, GPCRs may require desensitization through phosphorylation of G protein receptor kinases (GRKs) or binding of GPCR inhibitory proteins like arrestin (Venkatakrishnan et al., 2013; Syrovatkina et al., 2016). GNAQ’s specific GPCR interaction type is unknown at this time, and additionally, it is possible the GPCR-ligand state is less relevant under GNAQ* constitutive activity.

G protein coupled receptor extracellular ligands are divided into three categories: agonist, inverse agonists, and antagonists. Agonists bind to GPCRs and promote G protein signal transduction while inverse agonists stabilize the inactive “off” state. Antagonists do not change the equilibrium dynamics of the GPCR’s active and inactive conformations but do block binding of agonist and inverse agonist ligands.

All four Gα_*q*_ family members are palmitoylated at the Cys^9^ and Cys^10^ amino acid residues, but this post-translational modification is not well understood because it does not seem to affect association of GNAQ with Gβγ at the GPCR interface, nor downstream effector functions of GNAQ, such as activation of the phospholipase C pathway (Hepler et al., 1996).

### GNAQ regulation

G proteins like GNAQ can be regulated by a variety of intracellular proteins (besides GPCRs), including Ric-8 (synembryn), G-protein regulatory (GPR)-domain containing proteins (GRPs), Gα-binding and activating (GBA) motif-containing proteins, and regulators of G-protein signaling (RGS) proteins.

Mammals have both Ric-8A and Ric-8B and both have been demonstrated to interact with GNAQ. Ric-8A is widely expressed while Ric-8B is restricted mostly to olfactory tissue. Ric-8A is a GEF that promotes GDP release and stabilizes the nucleotide-free GNAQ transition state by initiating conformational changes to Switch I and II regions, thereby exposing the GTP binding site to the solvent, leading to formation of the GNAQ-GTP complex (Tall et al., 2003; Tall and Gilman, 2004). The lack of crystal structures prevents resolution of this exact mechanism, but it is interesting that Ric-8 acts on the GNAQ-GDP monomer itself, rather than the whole GNAQ-GDP/Gβγ complex (like GPCRs), as well as in concert with other regulatory proteins like GRPs. Additionally, Ric-8 plays other roles as a chaperone during Gα folding and processing in addition to membrane translocation (Gabay et al., 2011).

G-protein regulatory-domain containing proteins contain an ∼25 amino acid long G protein regulator domain, also called a GoLoco domain, that prolongs signal transduction by sequestering Gα from the Gβγ complex and possibly assisting Ric-8A activation of the GNAQ subunit (Kimple et al., 2002).

Gα-binding and activating domain-containing proteins, like Girdin, Dapple, NUCB1/2, and GBAS-1 interact with Gα proteins to initiate Akt signaling by accelerating the exchange rate of GDP (Garcia-Marcos et al., 2009; Ghosh et al., 2011). As rheostats, these GBA proteins have a powerful ability to fine-tune the duration of signaling and have been implicated in cancer progression and metastasis (Ghosh et al., 2011). GBA-domian proteins bind to Gα_*i*_ and Gα_*s*_ family members, but their direct interactions with GNAQ have yet to be confirmed.

Lastly, RGS modulate the intrinsic GTPase activity of the Gα subunit by stabilizing the GTP hydrolysis transition state, thereby encouraging deactivation of Gα. Heterogeneity in the RGS domains leads to preferential selectivity between RGS proteins and Gα subunits due to sequence-specific interactions between RGS domains and the Gα Switch I, III, and N-terminal side of Switch II (Baltoumas et al., 2013). RGS1, 3, 4, 8, 16, 17, and 18 bind to GNAQ and other Gα members; RGS2 seems to be specific to GNAQ (Soundararajan et al., 2008). RGS proteins may be an attractive target for drug development for a variety of diseases, including SWS (Sjögren, 2011).

### GNAQ signaling pathways

There are a variety of well-defined downstream targets of G proteins, although their role in PWB is not understood. GNAQ in particular can stimulate the phospholipase C β (PLC-β) isoforms through interactions with the GNAQ N-terminal β-1 strand, which cleaves phosphatidyl inositol 4,5-bisphosphate into inositol triphosphate (IP3) and diacylglycerol (DAG) (Hepler et al., 1996; Rhee and Bae, 1997; Baltoumas et al., 2013). IP3 opens the calcium channel IP3 receptor on the endoplasmic reticulum (ER) membrane while DAG activates protein kinase C (PKC). Additionally, GNAQ uses helix-loop-helix domains in Switch II and α-helix 3 to interact with RhoGEFs like p63RhoGEF through p63RhoGEF Dbl-homology/pleckstrin-homology (DH/PH) domains (Lutz et al., 2007; Baltoumas et al., 2013; Syrovatkina et al., 2016). Less well defined targets of GNAQ signaling include GRK2, actin, tubulin, PI3K, TPR1, Btk tyrosine kinase, phospholipase C-ε, and TRPM8 (Syrovatkina et al., 2016). The biological significances of these are not well understood and may lie in tissue-specific functions. It is unclear how EC GNAQ* affects these factors.

It is important to note there are many Gβγ interactions that are influenced by GNAQ activation, including adenylate cyclase, PI3K, potassium and calcium channels, and possibly IP3 receptors, Raf kinases, protein kinase D, histone deacetylase 5 (HDAC5), tubulin, F-actin, vinculin, ElmoE, Rab11, mitofusinil, Radil, activator protein 1, TFE3, and TRPM1 (Syrovatkina et al., 2016).

There are no therapeutic agents used clinically to target GNAQ protein at the time of this manuscript’s publication (Musi et al., 2019). Instead, many treatment strategies (discussed earlier) either manage symptoms or in the case of cutaneous lesions, aim to target pathways that limit vessel regrowth after laser treatment.

## Overlap with other vascular malformations

GNAQ mutations are found in a variety of diseases, including but not limited to: p.T96S NK/T cell lymphoma and diffuse bone and soft tissue angiomatosis (Li et al., 2019; Gaeta et al., 2020); p.R183Q Klippel-Trenaunay syndrome (He et al., 2020); p.V179M and p.F335L dark skin point mutations and hyperpigmentation (Garcia et al., 2008; Van Raamsdonk et al., 2009; Jain et al., 2020); p.Q209L or silencing mutations in non-small cell lung cancers (Choi et al., 2020); p.D663fs insertion and p.R385* nonsense mutations in melanocytoma (Francis et al., 2016); p.Q209L and p.Q209P intramedullary and leptomeningeal melanomas (Fortin Ensign et al., 2020); p.S12fs*49 breast cancer (Schrader et al., 2016); and decreased GNAQ expression in brain aging and neurodegeneration (Wettschureck et al., 2005; Frederick et al., 2012; Chen et al., 2017; Arey et al., 2018; Zhai et al., 2019; Sun et al., 2020). The following diseases discussed in more detail were selected due to the possible overlap in pathogenesis or molecular interactions, with the goal of spurring synergistic research encompassing these conditions and SWS.

### Brain arteriovenous malformations

Understanding the molecular etiology of PWBs may benefit from overlapping research conducted in brain arteriovenous malformations (bAVMs). bAVM form when afferent arteries abnormally communicate directly with draining veins without an intermediary capillary bed, sometimes in a tangle called a nidus. Like PWB/SWS, bAVMs can differ substantially in size, location, morphology, architecture, presentation, and clinical treatment/management between patients (Abecassis et al., 2014). The rate of detection is 1.12–1.42 per 100,000 person years. Unlike SWS, hemorrhage is the most common presenting characteristic of bAVMs, occurring in about half of new diagnoses, with larger nidus size and deep location significantly correlated with hemorrhage risk (Stefani et al., 2002; Halim et al., 2004; Abecassis et al., 2014). Seizures (30% of new diagnoses) and headaches (5–14%) are also detected. bAVMs account for a quarter of hemorrhagic stroke in adults under 50 and almost half of bAVM patients die or have significant impairments within a year of a hemorrhagic event (van Beijnum et al., 2009; Cordonnier et al., 2010).

Next-gen DNA sequencing of sporadic bAVM has identified KRAS somatic activating mutations localized to the EC compartment in about half of patients (Barbosa Do Prado et al., 2019). These KRAS mutations activate MAPK-ERK and PI3K-AKT pathways that increase phospho-ERK levels and lead to overall increases in EC angiogenesis, aberrant vascular EC cadherin localization, Notch signaling, and migration capacity; countering ERK overactivity with trametinib inhibition of MEK reversed these findings (Al-Olabi et al., 2018; Nikolaev et al., 2018). These data support the idea that MAPK-ERK activation drives bAVM formation in KRAS mutant spontaneous bAVM in a manner similar to studies that identify KRAS overactivation in PWB. Additionally, zebrafish bAVM models treated with the BRAF inhibitor vemurafenib had restored blood flow compared to their untreated controls (Al-Olabi et al., 2018).

TGFβ family signaling mutations in familial bAVMs are detected in the autosomal dominant disease hereditary hemorrhagic telangiectasia (HHT), which affects roughly 1 in 5,000 people and is characterized by AVM formation and hemorrhage in multiple organs, including the brain, lungs, liver, and/or gastrointestinal tract (Barbosa Do Prado et al., 2019). EC ENG (HHT type 1) and ALK1 (HHT type 2) mutations make up the vast majority of HHT cases, while a small number can be attributed to mutations in SMAD4. In the absence of functional Alk1 it appears that angiogenic and/or inflammatory signals subsequent to injury are required for bAVM formation, and this likely involves VEGF-stimulated angiogenesis (Chen et al., 2013). Interestingly, localized VEGF delivery in combination with ALK1 or ENG deficiency in EC, but not vascular smooth muscle cells, pericytes, or macrophages was required for bAVM formation in adult mice, suggesting that EC might be intrinsically responsible for the formation of bAVM networks (Choi and Mohr, 2005; Chen et al., 2013; Cheng et al., 2019). Others have proposed similar EC-stroma miscommunication in SWS disease pathology; although SWS dilated vessels are unlike high-risk hemorrhagic lesions seen in HHT and bAVM, similar deficiencies in mural cell recruitment and wrapping as well as the presence of pro-inflammatory immune infiltration may exacerbate vessel dilation and fibrosis, possibly through the same mechanisms (Chen et al., 2008, 2013; Couto et al., 2016).

As noted above, aberrant angiogenic control is implicated in bAVM progression. EC deficiencies in TGFβ family signaling can cause bAVM formation through several mediating factors, including loss or depletion of SMAD4, or activation of MEK/ERK (Ola et al., 2016; Kim et al., 2018). Lastly, reduced expression of integrin β8 (ITGβ8) in bAVM promotes hemorrhage in response to VEGF stimulation, possibly through increased Notch and Sox-2 (Su et al., 2010; Yao et al., 2019). Together, these results suggest that both ITGβ8 and ALK1 are crucial for maintaining a healthy pro- versus anti-angiogenic balance. VEGF-A antagonism with bevacizumab or pazopanib can restore the angiogenic balance and prevent lesion formation (Walker et al., 2012). Further work in this area is required, especially as overlapping research in MEK/ERK activation in SWS and bAVM may synergize.

Vessel immaturity and structural defects of the bAVM vascular wall are linked to PDGF/PDGFRβ deficiency. Dilated vessels with diameters greater than typical capillaries (greater than 15 μm) are strongly correlated with decreased smooth muscle cell and pericyte coverage and increased vascular permeability and hemorrhage risk (Chen et al., 2013; Zhu et al., 2018; Winkler et al., 2019; Pan et al., 2021). At this time, it is unclear if expression causes or results from bAVM formation, but increasing pericyte recruitment in the bAVM nidus by PDGFB expression or thalidomide/lenalidomide treatment reduces future hemorrhage risk (Lindahl et al., 1997; Lebrin et al., 2010; Zhu et al., 2018). Increasing pericyte coverage in PWB lesions may help prevent progressive vessel dilation and instability.

Non-coding regulatory RNAs may also play a role in vascular malformation development and/or progression. Downregulation of nicotinamide adenine dinucleotide phosphate (NADPH) reductase and lipoprotein lipase *via* several long non-coding RNAs may be associated with patient seizures in bAVM, while dysregulated novel miRNAs have been described to affect VEGF signaling and smooth muscle cell behavior (Huang et al., 2017; Li et al., 2018; Cheng et al., 2019). Inactivating mutations in DROSHA miRNA processing machinery have also been characterized in zebrafish bAVM models and have been identified in HHT patients that lack other known AVM-causative mutations (Jiang et al., 2018). To the best of our knowledge, miRNA dysregulation or misprocessing have not been examined in PWB/SWS and warrant investigation.

### Non-cutaneous melanoma

GNAQ p.Q209 mutations have been implicated in more than 90% of cases of GNAQ-driven uveal melanoma (UM), although, studies conflict regarding the downstream molecular interactions driving cancer formation and progression (Van Raamsdonk et al., 2009; Feng et al., 2019; Musi et al., 2019; Truong et al., 2020). The p.Q209P mutation is fairly common in UM and does not appear to significantly alter disease prognosis compared to p.Q209L, while the p.R183Q mutation is slightly less common and leads to less aggressive disease (Daniels et al., 2012; van Weeghel et al., 2019). The p.Q209* mutations are predicted to cause more severe effects through overactivation of the MAPK pathway (Schneider et al., 2019; Jain et al., 2020). GNAQ p.G48L mutation in UM is rare (Krebs et al., 2020).

Uveal melanoma is currently treated with surgical resection or radiation therapy at the primary tumor site, however, treatment has not improved in decades and about half of UM is diagnosed at a late stage after metastasis has already occurred (Pyrhönen, 1998; Rietschel et al., 2005; Daniels et al., 2012; Feng et al., 2019; Musi et al., 2019; van Weeghel et al., 2019). Feng et al. have demonstrated that the presence of oncogenic GNAQ mutations can cause an overactivation of FAK-mediated YAP activation, which transcriptionally activates the TEAD transcription factor family and downstream pro-growth and pro-survival genes (Feng et al., 2019; Truong et al., 2020). The tumor suppressive Hippo pathway is not able to effectively silence this cascade during constitutive GNAQ activity (Feng et al., 2019). No specific YAP inhibitors are currently in clinical use, however, Truong et al. demonstrated that combined therapy with trametinib MEK1/2 inhibition and the lysosome inhibitor chloroquine increased cytotoxicity while indirectly decreasing YAP nuclear localization and transcriptional activity (Feng et al., 2019; Truong et al., 2020). Other groups have proposed GNAQ overactivation of ERK1/2 or MEK1/2 as a driver of UM, but inhibitors of this pathway alone are typically insufficient to stop progression and the degree of MAPK activation is widely heterogeneous within tumor sites (Zuidervaart et al., 2005; Boru et al., 2019; Feng et al., 2019; Musi et al., 2019; Truong et al., 2020).

Musi et al. (2019) demonstrated a dose-dependent reduction in mutant GNAQ-driven activation of ADP-ribosylation factor 6 (ARF6) using tris dibenzylideneacetone (DBA) palladium. Moreover, ARF6 GTPase has been identified as an immediate downstream cancer driver in GNAQ-UM, where it plays a role in the localization and transactivation of β-catenin from the plasma membrane to the nucleus, as well as the activation of Rho-Rac pathway signaling (Yoo et al., 2016; Musi et al., 2019). Other groups have demonstrated that tris DBA compounds decrease MAPK, PKC, and AKT-driven cancers as a result of NMT-1 blockade, however Musi et al. (2019) did not observe suppression of these pathways nor FAK inhibition and, interestingly, saw an increase in ERK and AKT phospho-activation despite increased UM apoptosis. Gene array analysis suggests tris DBA palladium additionally interferes with tumor RNA splicing and reduces resistance to chemotherapy (Musi et al., 2019). Many of these factors driving UM may be drivers of PWB/SWS.

The presence of a nevus of Ota is one of the few predictors of risk for UM and Van Raamsdonk et al. (2009) have identified an 83 and 46% incidence of GNAQ p.Q209L mutations in blue nevi and UM, respectively, underscoring the overlapping relationship GNAQ plays in melanocytic neoplasms and UM. On the other hand, approximately one in 400 nevi of Ota progresses to UM so other factors remain undefined. Van Raamsdonk et al. postulate that GNAQ downstream targets such as ERK- and endothelin-regulated developmental survival, as well as Wnt and metabotropic glutamate receptor (GRM1) signaling contribute to melanocytic neoplasia oncogenesis and metastasis (Van Raamsdonk et al., 2009; Jain et al., 2020). Additionally, transfection of GNAQ p.Q209L in melanocytes *in vitro* demonstrated unusual anchorage-independent growth as well as large, irregularly shaped nuclei (Van Raamsdonk et al., 2009).

### Cherry angiomas

Cherry angiomas are the most common kind of vascular tumor in adults. They typically present on the trunk or upper extremities as small, round, red to purple dome-shaped papules composed of dilated, thin-walled capillaries surrounded by hyalinized stroma, somewhat similar to the dilated structure of cutaneous PWB (Liau et al., 2019). Liau et al. (2019) detected EC GNAQ mutations in cherry angioma-like lesions in about half of all patients studied and are predicted to activate the MAPK pathway to drive pathogenesis, as in PWB/SWS. It is possible that significant overlap between cherry angiomas and PWB/SWS could offer us a glimpse of how PWB/SWS lesions form *in utero* and close collaboration between researchers working in the two diseases could afford greater understanding of both.

## Models of port-wine birthmark/Sturge-Weber syndrome

### *In vivo* models

Most *in vivo* studies are typically performed using healthy animal skin models such as mice, Wistar rats, or chicken (combs or wattles) where intervention is focused on regressing normal vessels (Rikihisa et al., 2017, 2018). Alternatively, xenotransplants can be used where mutant human EC in Matrigel extracellular matrix are subcutaneously injected into mice to study vessel formation, albeit outside the normal PWB/SWS environment. Most data to date identifying mutant GNAQ and possible molecular interactions leading to pathology have been developed using genome (DNA) sequencing and histology of patient tissue samples (Shirley et al., 2013; Nakashima et al., 2014; Couto et al., 2016; Huang et al., 2017; Bichsel and Bischoff, 2019; Le Guin et al., 2019; Lee et al., 2019; Cong et al., 2020; Jordan et al., 2020). Additional *in vivo* and *in vitro* complex studies like those performed by Huang et al. are needed to advance our understanding of PWB. The authors in this study identified Angpt2 as a downstream factor increased by endothelial GNAQ p.R183Q signaling that caused vessel dilation; this feature was reversed through GNAQ inhibition using YM-254890 or shRNA knockdown of Angpt2 (Huang et al., 2022). Thorough studies that identify direct controllers of PWB vessel dysfunction are rare.

### *In vitro* models

As noted, there is a critical need for better models of PWB and SWS due to an absence of naturally occurring GNAQ* PWB/SWS in non-human animals and an inability of engineered *in vivo* models to successfully recapitulate SWS disease biology of humans. *In vitro* models may meet this need. Unfortunately, *in vitro* monolayer studies are inadequate for studying the complex disease pathology of PWB, which involves multiple tissues and cell types and is characterized by clearly three dimensional lesions – dilated blood vessels (Sun et al., 2020; Ewald et al., 2021). Complex human disease models such as microphysiological systems (MPS) provide an exciting and untapped opportunity (Ewald et al., 2021). Frequently referred to as an organ-on-a-chip, these platforms combine relevant cell types in a three-dimensional, ECM-rich tissue bed, often with media delivered to the tissue chamber by perfusable EC-lined microvessels (Wang et al., 2016, 2017; Ewald et al., 2021; Yue et al., 2021). Phan et al. and others have demonstrated the utility of these platforms for screening novel and repurposed therapeutics (Sobrino et al., 2016; Phan et al., 2017; Liu et al., 2020; Hachey et al., 2021). In particular, MPS platforms hold promise for rare diseases like SWS, as they can successfully recapitulate many aspects of disease biology and they offer the ability to rapidly test molecular perturbations, such as gene deletion or replacement. To the best of our knowledge, no complex *in vitro* model of PWB/SWS has been published. We argue that MPS technology holds great promise for understanding the GNAQ* downstream effectors in disease progression.

In a recent manuscript posted on BioRivx, Soon et al. (2022) describe a mosaic EC KRAS^*G*12*V*^ MPS of sporadic bAVM. The authors were able to detect increased vessel dilation and permeability in KRAS^*G*12*V*^ EC due to breakdown in adherens junctions and increased VEGF signaling. Finally, the authors were able to reverse some of this pathology with pharmaceutical MEK inhibition but not PIK3 inhibition, supporting further investigation in the clinic of MEK inhibition in vascular malformation studies. Complex *in vitro* studies of this nature are needed to understand PWB/SWS pathology.

## Discussion

Sturge-Weber syndrome is a complicated disease in which GNAQ mutations drive progressive capillary vessel dilation and dysfunction in skin, eye, and brain tissue. The consequences for these CMs entail severe impacts to patient quality of life and include but are not limited to: glaucoma, epilepsy, cognitive deficits, and psychosocial ramifications.

As noted, in contrast to several vascular malformations, there are few good models for PWB/SWS, and as a result there are still many questions left unanswered about how PWB/SWS arises and progresses. Are the effects of GNAQ* EC autonomous or is additional dysfunction in the stroma required? How do we reconcile these disease models with the rare contribution of additional GNAQ* in non-EC cell types? What are the critical downstream effectors of GNAQ that cause disease progression, and can they be specifically targeted in affected skin, brain, and eye tissue? We desperately need better models that evaluate transcriptional changes in PWB/SWS to answer these questions and to provide platforms for therapeutic drug development.

## Author contributions

WVT performed the literature review and wrote the manuscript with input from all authors. KK and CH supervised assembly of the manuscript and provided critical feedback.
